# Chemical and nutritional properties of channel and hybrid catfish byproducts

**DOI:** 10.1002/fsn3.483

**Published:** 2017-05-17

**Authors:** Peter J. Bechtel, John M. Bland, Karen L. Bett‐Garber, Casey C. Grimm, Suzanne S. Brashear, Steven W. Lloyd, Michael A. Watson, Jeanne M. Lea

**Affiliations:** ^1^ USDA‐ARS Southern Regional Research Center Food Processing and Sensory Quality Unit New Orleans LA USA

**Keywords:** Catfish byproducts, channel catfish, fish byproducts, hybrid catfish

## Abstract

The objective of this study was to chemically characterize both channel and hybrid catfish parts including heads, frames, viscera, skin, and fillet trimming mince. Triplicate samples of channel and hybrid catfish byproduct parts were obtained from a large commercial catfish processor and analyzed for percent moisture, lipid, protein, ash, and amino acid and fatty acid profiles were determined. The content of the off‐flavor compounds, 2‐methylisoborneol (MIB) and geosmin were also determined. The lipid content of samples were 13.6% and 10.0% for channel and hybrid skins, 17.7% and 21.4% for channel and hybrid viscera, 20.0% and 19.1% for channel and hybrid frames, and 9.7% and 9.3% for channel and hybrid heads. The protein content of samples ranged from a high of 22.8% for channel catfish skins, to a low of 13.4% for channel frames. Low levels of geosmin, <1 ppb, were detected in the byproduct samples, while no MIB was detected. Palmitic, oleic, and linoleic acid comprised approximately 80% of the fatty acids in the byproduct tissues. The amino acid profiles indicated that the catfish mince had high levels of lysine and methionine and other essential amino acids. Results from this study will be used in the development of new value‐added products from catfish byproducts.

## 1 Introduction

Processing of catfish results in the production of a large amount of fish waste. In 2014, 136,975 metric tons of catfish was processed in the US (Hanson, 2015). Depending on what product is being produced, the waste (or byproduct) can account for greater than 60% (82,000 metric tons in 2015) of the harvested weight of the fish. Fish processing byproducts consist primarily of heads, viscera, frames, skin, and lesser amounts of blood and fins (Crapo & Bechtel, 2003). Currently catfish byproduct from commercial processing operations is combined and sold to rendering plants where the byproduct is made into protein meals and oils used primarily as feed ingredients. However, some smaller operations may choose to dispose off the waste, make fertilizers, or use it directly as a feed ingredient. In addition to conventional rendering of catfish waste there are two catfish byproduct processing plants, one of which uses a new drying technology to make a catfish meal and oil (Gresham, 2012) and the other a fertilizer by hydrolyzing catfish byproducts. Due to the large increases in prices for fish meal and fish oil during the last decade, raw fish byproduct has a significant if unrealized value (Bechtel & Smiley, 2010). There are few US catfish processors that manufacture other products from their catfish waste. Little use has been made of individual parts such as skin, or viscera components such as stomachs and livers, to make value‐added products.

Other major aquaculture industries and wild fish processors are devising ways of getting more profit from their fish byproduct (Bechtel, 2003a; Bechtel & Smiley, 2010; Shahidi, 2007). Some progress has been made in evaluating catfish byproduct components such as using fish skin for making gelatin (Jiang, Shaoyang, Du, & Wang, 2010; Yang et al., 2007; Yin, Pu, Wan, Bechtel, & Sathivel, 2010), Vietnamese catfish meals (Nguyen, Lindberg, & Ogle, 2007), catfish oil (Sathivel, Prinyawiwatkul, Grimm, King, & Lloyd, 2002), catfish oil extraction (Sathivel, Yin, Prinyawiwatkul, & King, 2009b), catfish protein and hydrolysates (Davenport & Kristinsson, 2011; Theodore, Raghavan, & Kristinsson, 2008; Yin, Wan, Pu, Bechtel, & Sathivel, 2011), mince from frames (Hoke, Jahncke, Silva, Hernsberber, & Suriyaphan, 2000), minced belly flap meat (Wiles, Green, & Bryant, 2004), and catfish roe (Sathivel, Yin, Bechtel, & King, 2009a).

To increase the value of byproduct, it is imperative to know the composition of the raw material produced using modern automated processing equipment such as has been reported for other species (Bechtel, 2003a; Bechtel et al., 2009; Gunasekera, Turoczy, De Silva, & Gooley, 2002; Kotzamanis, Alexis, Andriopoulou, Castritsi‐Cathariou, & Fotis, 2001; Oliveira, Bechtel, Lapis, Ellingson, & Brenner, 2011; Oliveira, Bechtel, Morey, Lapis, & Brenner, 2014). The reference by Bryan, Freeman, and Graci (1979) provides limited information on selected channel catfish byproducts. However, there is little literature that characterizes both channel and hybrid catfish byproducts obtained from mechanized commercial catfish processing. Byproducts that are produced during mechanical processing of many species of fish include heads, frames, skin, viscera, and mince (fillet trim). These byproduct parts reflect the common unit operations that include heading, removal of viscera, cutting of the fillets from the back bone, removal of skin from fillets, and fillet trimming. The US catfish industry is evolving with greater production of hybrid catfish. Hybrid catfish differ from channel catfish in having a higher fillet yield, smaller heads, less viscera and a greater percent of visceral fat (Bosworth, Wolters, Silva, Chamul, & Park, 2004). There is a lack of data on the composition of hybrid catfish byproducts.

The objective of this research was to determine the chemical composition of channel and hybrid heads, frames, skin, viscera, and mince. This research is part of an effort to increase the value of fish processing byproducts by enhancing the knowledge of the chemical composition of fish byproducts that show potential as raw materials for the production of specialty food and feed ingredients.

## 2 Materials and methods

### 2.1 Samples

Live catfish estimated to weigh between 0.45 kg and 0.9 kg were delivered on the mornings of December 6 (channel catfish) and December 11, 2012 (hybrid catfish) to a large commercial catfish processor in Mississippi. The processing line was totally automated with mechanical headers, extraction of viscera, separation of the fillets from the backbone, and removal of the skin from the fillet. Fillet mince was obtained from manually trimming of fillets prior to freezing or packaging. Three separate samples, 3–4 kg, of each catfish byproduct, including heads, frames, skin, viscera, and fillet mince were collected from the operating processing lines in plastic bags, which were placed in coolers and ice maintained in the coolers during transportation to the USDA/ARS Southern Regional Research Center in New Orleans, LA. Samples were then immediately frozen and held at −20°C until ground and analyzed. Samples were thawed and comminuted using a Hobart meat grinder (Model 548SS; Marblehead, OH) fitted with a plate with 6.4 mm holes. Half of each sample was stored at −70°C as raw sample and the other half was freeze dried (Virtis ES 35 Freeze Dryer) and then pulverized in a 1 L blender and the powder stored at −70°C. Frames and heads contain substantial amounts of skeletal muscle such as the collar area and cheek muscle tissue in heads, and some muscle tissue remains attached to the frame after the mechanical filleting operation. Catfish heads contained the heart and some of the liver. Frames also had some fins and some ribs attached. Viscera consisted of the stomach, intestine, internal fat pad, some liver and testes and egg when present. Skin contained small amounts of meat attached. Mince was predominantly pieces of muscle with some ribs and membrane components. Three replicate samples of the five body parts for both channel and hybrid catfish resulted in thirty samples.

### 2.2 Proximate analysis

Moisture and ash contents were determined using AOAC methods #952.08 and #938.08, respectively (AOAC, 1990). Nitrogen content was accessed by pyrolysis with a LECO FP‐2000 nitrogen analyzer (LECO Co., St. Joseph, MO). Protein content was calculated as 6.25 times % N. Total lipid content was determined gravimetrically by the method of Folch, Lees, and Sloane‐Stanley (1957) after extraction with an ASE (Dionex ASE 200) using methylene chloride. Solvent was removed under a N_2_ gas stream at 40°C using a TurboVap LV (Caliper Life Sciences) in preweighed vials. The remaining traces of solvent were removed under vacuum until constant weight was achieved, and percent lipids were determined gravimetrically. Oils were stored at −70°C until further analysis.

### 2.3 Preparation of fatty acid methyl esters and gas chromatography‐mass spectral analysis

Fatty acid methyl esters (FAME) were prepared using methanolic sodium methoxide in diethyl ether with methyl acetate for 15 min at ambient temperature, as described by Christie (1982). After quenching with saturated oxalic acid in ether, and centrifugation, the supernatant was evaporated to dryness in vacuo and FAMEs were dissolved in hexane for immediate analysis. Analysis was carried out on a GC model 6,890 with MS model 5973 (Agilent Technologies) fitted with an HP‐88 (30 m × 0.18 mm id., 0.18 μm film) limited thermal mass (LTM) capillary column (Agilent Technologies). The LTM column heating was controlled by integrated Gerstel Maestro software, beginning with a 1 min hold at 50°C, followed by a 25°C/min gradient to 140°C, a 3°C/min gradient to 215°C, and a 5°C/min gradient to 227°C. A helium flow rate of 1 ml/min was maintained by a programmed pressure gradient in the Chemstation software. An autosampler performed the GC injections of standards and sample, and injection volume was 1 μl. Data were collected and analyzed using the MSD ChemStation program (Rev.D.03.00.552; Agilent Technologies 1989‐2006). Identification of peaks was performed using a Supelco^®^ (Bellefonte, PA) 37 component FAME mix standard, using base peak mass quantification curves to calculate amounts and percent FAME.

### 2.4 Amino acid analysis

Amino acid profiles were determined by the AAA Service Laboratory Inc. (Boring, OR). Samples were hydrolyzed with 6 mol/L HCl and 2% phenol at 110°C for 22 hr. Amino acids were quantified using the Beckman 6300 amino acid analyzer (Beckman Coulter, Brea, CA) with postcolumn ninhydrin derivatization. To minimize methionine losses, oxygen was removed by evacuation of the hydrolysis tubes that contained samples and acid for 10 min prior to putting them in the hydrolysis oven. Cysteine and tryptophan were not determined as separate hydrolysis procedures are required, which increases analysis cost. Two samples of each type of tissue were analyzed for amino acid composition.

### 2.5 Geosmin and 2‐Methylisoborneol analysis

Catfish were analyzed following the procedure described in Grimm, Lloyd, Batista, and Zimba (2000) with modifications made due to different equipment. Tissue was chopped into small pieces and the 20.0 ± 0.05 g chopped tissue was placed in a modified vacuum trap (Widgett Scientific, Baton Rouge, LA, USA) and 5 μl of a 10 ppm aqueous solution of decahydro‐1‐naphthol (DHN) were added as an internal standard. The trap was placed in a modified 1,000 W microwave oven (Kenmore, Hoffman Estates, IL, USA). Two holes were cut in the oven and protected with aluminum foil cylinders. A microwave detector was used to assure that no significant microwave energy escaped the oven. Nitrogen flowed in through the top hole and steam and N_2_ flowed out the side hole into a 50 ml glass cylinder located in a cold bath (Lauda, model RM6, Konegshofen, Germany) held at 0.0°C. The oven was operated at 20% power for 6 min while the N_2_ flowed at 40 ml/min. After heating, deionized water was used to make the total volume 14 ml. Seven milliliter was pipetted into each of two vials containing 2.9 g sodium chloride and then sealed with Teflon‐lined screw caps (Sigma‐Aldrich, St. Louis, MO, USA).

Vials were placed in an autosampler (model MPS‐2, Gerstel, Baltimore, MD, USA) maintained at room temperature until analyzed. Each sample was heated to 65°C and agitation at 750 rpm for 5 min. Samples in vials were then exposed to the 1‐cm‐long divinylbenzene–carboxen–polydimethylsiloxane SPME fiber (Supelco, Bellefonte, PA) for a 15‐min adsorption period while being agitated at 250 rpm. The fiber was withdrawn from the vial and desorbed at 270°C for 1 min in the injection port of a HP6890 GC equipped with a 5,973 mass‐selective detector (Hewlett‐Packard, Palo Alto, CA). The injection port was operated in pulsed‐splitless mode and fitted with a 0.75‐mm‐i.d. injection liner. The head pressure was set to 50 psi of helium for the first minute and then to a constant velocity of 40 cm/s for the remainder of the GC run. The quadrupole MS was operated in electron ionization mode. Selected Ion Monitoring (SIM) was employed for quantification of the target and qualifier ions for MIB, geosmin, and DHN.

### 2.6 Statistical analysis

The weighted means were derived from an analysis of variance (ANOVA) and the mean comparison test, Tukey‐Kramer adjustment to Least Squares Means, were performed in Proc Mixed using Enterprise Guide, version 5.1, (SAS, Inc., Cary, NC). Significance was reported at *p* < .05 for all data.

## 3 Results and discussion

### 3.1 Proximate analysis

The moisture, protein, lipid, and ash contents of the five byproduct parts for both channel and hybrid catfish are listed in Table 1. Comparisons of common parts between channel and hybrid catfish indicate that there are significant differences between the composition of frames and skin for percent moisture, protein, and fat, although the numerical differences were small.

**Table 1:** Proximate composition of channel and hybrid catfish byproducts

	Channel					Hybrid				
	Frame	Head	Skin	Mince	Viscera	Frame	Head	Skin	Mince	Viscera
% Moisture	59.50^bZ^	68.29^a^	65.65^aZ^	64.68^a^	67.80^a^	61.62^cY^	68.23^ab^	70.40^aY^	63.01^bc^	66.17^abc^
SD	0.69	0.29	0.96	3.98	2.37	0.39	0.57	1.18	3.27	3.68
% Ash	5.09^bY^	6.70^a^	0.59^d^	2.25^c^	1.04^dY^	4.58^bZ^	6.99^a^	0.57^d^	1.93^c^	0.76^dZ^
SD	0.1	0.32	0.31	0.58	0.03	0.19	0.29	0.24	0.23	0.05
% Lipid	20.03^aY^	9.74^c^	13.62^bY^	19.22^a^	17.65^a^	19.13^aZ^	9.33^b^	10.00^bZ^	21.58^a^	21.40^a^
SD	0.45	0.86	0.6	2.33	2.04	0.3	0.48	1.15	2.56	3.72
% Protein	16.35^bY^	15.80^b^	22.82^aY^	15.41^b^	13.35^c^	15.72^bZ^	15.99^b^	19.89^aZ^	14.45^c^	13.77^c^
SD	0.42	0.48	0.65	1.69	0.71	0.77	0.68	0.49	1.56	1.11

Values are means from three replicate samples and standard deviation.

a, b, c, d within a row and within species (channel or Hybrid) represent significant differences (*p* < .05) based on Tukey‐Kramer adjustment to least square means test.

Y, Z within a row but between common byproduct parts (channel frames vs. hybrid frames) represent significant differences (*p* < .05) based on Tukey‐Kramer adjustment to least square means test.

The moisture content ranged from a high of 70.4% for hybrid skin to a low of 59.5% of channel frames. As expected there were significant differences between the ash % of different byproduct parts. Ash contents of 6.7 and 7.0% were found in the heads of channel and hybrid catfish, respectively. These values are similar to the value reported for channel catfish heads of 8% by Bryan et al. (1979) but higher than the values of 4.6, 4.2, and 3.6% for Alaska pollock, pacific cod, and pink salmon (Bechtel, 2003b). The high percentage of ash in catfish heads is of interest as the head as a percent of the body weight was reported to be 24% for channel and 21.2% for hybrid catfish (Bosworth et al., 2004). The ash content of catfish frames was 5.1 and 4.6 percent for channel and hybrid frames, respectively. These values are higher than reported for several marine species (Bechtel, 2003b). Ash values of the skin, guts, and mince were lower than those determined for heads and frames, reflecting the lack of bone in these parts. The mince had an average ash content of 2.1%, which could be attributed in part to the rib bones trimmed from the fillets.

The protein content of all parts ranged from a low of 13.4% for channel viscera to a high of 22.8% for channel skin. Similar high protein values of 25% and 24.5% were reported for Alaska pollock and pacific cod skin samples (Bechtel, 2003b); however, these values were higher than the 17% value reported for catfish skin by Bryan et al. (1979). The protein values for the catfish byproduct parts were similar to values reported for parts from other marine species (Bechtel, 2003b).

The lipid content in all the parts of catfish byproducts was >9.0% and values ranged from an average low value of 9.5% for heads to an average high value of 19.6% for frames. Bryan et al. (1979) reported % fat values of 3% for catfish skin, 8% for heads, and 15–33% for viscera depending on the time of the year samples were collected. One possibility for the higher fat % found in the heads of this study could be due to the pieces of liver that remained attached to the head after the beheading operation. In this study, the samples were collected in late fall and the viscera fat content was 21.4% for the hybrid and 17.7% for the channel. Bosworth et al. (2004) reported that the viscera for channel and hybrid catfish contributed 12.3% and 11.7% of the weight of channel and hybrid catfish. The average lipid content of the catfish skin in this study was 11.8%, which was much higher than the 3% value reported for catfish skins by Bryan et al. (1979) and values of 0.4 and 0.3% reported for Alaska pollock and pacific cod (Bechtel, 2003b). The high values found in this study could possibly reflect the skinning process where more of the subcutaneous fat layer was retained on the skin after the skinning operation.

There were significant differences in the fat content of the different byproduct parts with the heads and skin having lower lipid contents than frames, viscera, and mince (Table 1). However, the percent lipid found in all the byproducts was high when compared to the lipid levels found in ocean‐harvested marine byproducts. Catfish byproducts are a good source of fish oil and protein, which make these products good candidates for the production of food and feed oil and protein ingredients.

### 3.2 Fatty acid profiles

Oils were extracted from all channel and hybrid catfish byproducts and the fatty acid methyl ester (FAME) profiles are shown in Table 2. Quantities of FAME (above 1%) are reported in the table. The most abundant FAME in all catfish tissues evaluated was oleic acid, where values ranged from a high of 45.6% in hybrid mince to a low of 40.6% in hybrid heads. The second most abundant fatty acid was linoleic ranged from 21.6% to a low of 17.6%. The third most abundant fatty acid was palmitic acid, which ranged from a high of 20.7% to a low of 17.4%. These three fatty acids constituted approximately 80% of the fatty acids found in all byproduct parts analyzed. Another fatty acid found in relatively high abundance in catfish parts was stearic acid, which ranged from 3.1% in hybrid skin to 4.6% in channel heads. The fatty acid compositions of channel and hybrid parts were similar.

**Table 2:** FAME profile (g/100 g oil) of channel and hybrid catfish byproducts

Fatty acids	Channel					Hybrid				
	Frame	Head	Skin	Mince	Viscera	Frame	Head	Skin	Mince	Viscera
C14	1.03	0.97^Z^	1.09	1.10	1.15	1.10	1.23^Y^	1.12	1.10	1.13
SD	0.10	0.04	0.11	0.04	0.17	0.13	0.03	0.04	0.02	0.02
C16	18.17^ab^	19.16^a^	17.57^abZ^	17.70^ab^	17.42^bZ^	18.33^ab^	20.69^a^	18.46^abY^	17.68^ab^	18.60^abY^
SD	0.48	1.32	0.31	0.08	0.20	0.55	2.04	0.10	0.15	0.09
C18	4.01	4.55	3.67	3.71	4.04	3.51^ab^	4.49^a^	3.07^b^	3.67^ab^	4.02^ab^
SD	0.66	1.06	0.47	0.14	0.66	0.38	0.55	0.07	0.44	0.46
Σ S FA	23.21	24.69	22.33	22.51	22.61^Z^	22.94^b^	26.41^a^	22.65^b^	22.45^b^	23.75^abZ^
SD	0.27	2.42	0.49	0.04	0.52	0.71	2.63	0.18	0.27	0.54
16:1ω7	2.37	1.88^Z^	2.60	2.51	2.86	2.54	2.41^Y^	2.70	2.50	2.53
SD	0.39	0.14	0.31	0.06	0.71	0.35	0.20	0.11	0.02	0.03
18:1ω9	43.25^bc^	42.60^c^	44.67^abcY^	45.48^a^	44.93^abY^	42.99^b^	40.63^c^	43.22^bZ^	45.58^a^	41.07^cZ^
SD	0.38	1.36	0.80	0.53	0.82	1.01	0.33	0.49	0.20	0.64
20:1ω9	1.28^bc^	1.36^aZ^	1.30^ab^	1.20^cZ^	1.26^bc^	1.29^b^	1.58^aY^	1.41^ab^	1.36^bY^	1.31^b^
SD	0.04	0.04	0.01	0.02	0.03	0.12	0.03	0.07	0.04	0.05
Σ M FA	46.90^ab^	45.84^b^	48.57^ab^	49.20^a^	49.05^aY^	46.81^b^	44.61^c^	47.33^b^	49.44^a^	44.91^cZ^
SD	0.05	1.45	1.08	0.58	1.54	0.56	0.45	0.61	0.21	0.67
18:2ω6	20.88	19.68	19.44	19.37	17.62	20.87^ab^	19.03^b^	20.71^ab^	19.43^ab^	21.58^a^
SD	0.23	1.23	1.55	0.73	2.98	0.43	1.83	0.40	0.33	0.12
18:3ω3	1.46	1.24	1.73	1.57	1.80	1.56	1.49	1.62	1.53	1.83
SD	0.21	0.08	0.26	0.12	0.40	0.19	0.24	0.05	0.07	0.02
20:2ω6	1.04	1.05	1.02^Z^	1.00	1.01^Z^	1.08^b^	1.13^ab^	1.11^abY^	1.06^b^	1.18^aY^
SD	0.11	0.08	0.03	0.03	0.07	0.03	0.01	0.03	0.03	0.01
20:3ω6	1.17	1.37	1.11	1.11	1.24^Y^	1.22	1.26	1.28	1.12	1.08^Z^
SD	0.07	0.13	0.18	0.03	0.05	0.07	0.16	0.06	0.08	0.05
22:6ω3					1.16					
SD					0.54					
Σ P FA	24.55	23.34	23.30	23.05	22.82	24.73^ab^	22.91^b^	24.72^ab^	23.14^ab^	25.67^a^
SD	0.17	1.52	1.49	0.63	2.12	0.31	2.19	0.49	0.26	0.18
Σ All FA	94.65^ab^	93.88^b^	94.20^ab^	94.75^a^	94.49^ab^	94.48^ab^	93.94^b^	94.70^a^	95.03^a^	94.32^ab^
SD	0.09	0.33	0.5	0.07	0.43	0.56	0.01	0.01	0.21	0.31
Σ ω3	1.46^b^	1.24^b^	1.73^b^	1.57^b^	2.96^a^	1.56	1.49	1.62	1.53	1.83
SD	0.21	0.08	0.26	0.12	0.94	0.19	0.24	0.05	0.07	0.02
Σ ω6	23.10	22.10	21.56	21.48	19.87	23.17	21.42	23.10	21.61	23.84
SD	0.17	1.44	1.74	0.74	3.06	0.49	1.96	0.47	0.24	0.18

If a given FA value was less than 1 g/100 g oil for all samples, the FA was not included in the table.

Values are means from three replicate samples and standard deviation.

S is saturated, M is monounsaturated, P is polyunsaturated, FA is fatty acid.

a, b, c, d within a row and within species (channel or Hybrid) represent significant differences (*p* < .05) based on Tukey‐Kramer adjustment to least square means test.

Y, Z within a row but between common byproduct parts (channel frames vs. hybrid frames) represent significant differences (*p* < .05) based on Tukey‐Kramer adjustment to least square means test.

Hybrid viscera had the highest levels of polyunsaturated fatty acids (PUFA) at 25.7%. The most abundant polyunsaturated fatty acid was linoleic. Values for arachidonic acid (20:4ω6) were below 1% in the byproducts and were not reported. The total omega‐3 values for catfish byproducts are lower than comparable values reported by Oliveira and Bechtel (2005) for pollock (25.4–35.3%) and salmon (27.8–35.2%) byproducts. The percent of the long chain omega 3 eicosapentaenoic acid (EPA) and docosahexaenoic acid (DHA) values were all less than 1% in all channel and hybrid byproducts. The only long chain omega‐3 fatty acid with a content of EPA or DHA over 1% was 22:6ω3 in channel viscera. Levels of monounsaturated fatty acids (MUFA) in catfish byproducts ranged from 49.4% for hybrid mince to 44.6% hybrid heads, which were higher than comparable values from pollock (34.0–47.1%) and salmon (34.2–42.7%) byproducts (Oliveira & Bechtel, 2005). The saturated fatty acids values for channel and hybrid byproduct parts are listed in Table 2. Percent saturated fatty acids were similar for comparable parts and ranged from 26.4% for hybrid heads to 22.3% for channel skin.

The P/S ratios ranged from 1.1 for hybrid viscera to 0.9 for hybrid head. The total omega‐3 content of fatty acids ranged from 3.0 to 1.2%, while the omega‐6 content ranged from 23.8% to 19.9%. Others have reported the omega‐3/omega‐6 ratio in fish byproducts (Gunasekera et al., 2002; Kotzamanis et al., 2001; Oliveira & Bechtel, 2005).

### 3.3 Amino acids profile

Table 3 lists the amino acid composition of channel and hybrid catfish parts including heads, frames, skin, viscera, and fillet mince. Few differences were detected between comparable channel and hybrid parts. Differences were determined between byproducts both for essential and for nonessential amino acids. Value for three potentially limiting amino acids ranged from 1.7 to 2.9% for methionine, 4.2 to 8.2% for lysine, and 3.1 to 5.0% for threonine. Methionine, lysine, and threonine were lower in skin than the other byproducts, reflecting the high collagen content in skin. Overall, the ranges for these three amino acids matched closely with results reported by Bechtel and Johnson (2004) for the amino acid composition of similar byproduct parts from pink salmon, Alaska pollock, and Pacific cod. In this study, values for cysteine and tryptophan were not determined due to increased analysis costs.

**Table 3:** Amino Acid Profiles of Channel and Hybrid Byproducts (% wt/wt)

	Channel					Hybrid				
	Frame	Head	Skin	Mince	Viscera	Frame	Head	Skin	Mince	Viscera
ALA	7.17^b^	7.88^b^	9.08^a^	6.85^b^	6.81^b^	7.47^b^	7.39^b^	8.83^a^	6.77^c^	6.51^c^
SD	0.19	0.13	0.11	0.44	0.48	0.01	0.16	0.02	0.09	0.11
ARG	6.87^b^	7.15^b^	8.03^a^	6.88^b^	6.83^b^	7.09^b^	6.98^bc^	8.10^a^	6.69^d^	6.79 ^cd^
SD	0.02	0.01	0.04	0.19	0.29	0.11	0.09	0.05	0.01	0.04
ASP	9.37^ab^	8.22^bc^	6.75^c^	10.32^a^	9.28^ab^	8.83^bc^	8.54^c^	6.59^d^	9.76^a^	9.50^ab^
SD	0.48	0.02	0.12	0.63	0.35	0.04	0.24	0.05	0.17	0.37
GLU	13.06^ab^	11.30^bc^	10.05^cY^	14.25^a^	12.33^abc^	12.05^b^	11.85^b^	9.66^cZ^	12.97a	11.61^b^
SD	0.69	0.31	0.13	1.14	0.29	0.25	0.18	0.01	0.04	0.37
GLY	11.24^bc^	15.30^ab^	20.19^a^	8.71^c^	9.26^c^	13.11^b^	13.47^b^	19.65^a^	9.64^c^	10.35^c^
SD	0.77	0.51	0.49	2.71	0.43	0.10	0.80	0.14	0.58	0.27
HIS*	1.84^b^	1.75^b^	1.17^c^	1.96^ab^	2.27^a^	1.89^c^	1.93^c^	1.30^d^	2.06^b^	2.24^a^
SD	0.05	0.08	0.06	0.19	0.04	0.01	0.00	0.00	0.04	0.06
Hlys	0.35^bcZ^	0.47^abZ^	0.64^aZ^	0.18^cZ^	0.27^bcZ^	0.85^Y^	0.99^Y^	1.35^Y^	0.91^Y^	1.12^Y^
SD	0.04	0.08	0.02	0.10	0.05	0.00	0.01	0.02	0.03	0.38
HYP	3.30^bc^	5.00^ab^	7.39^a^	2.13^c^	2.21^c^	3.99^b^	4.16^b^	7.00^a^	2.52^c^	2.60^c^
SD	0.50	0.30	0.24	1.24	0.44	0.09	0.39	0.01	0.32	0.08
ILE*	3.77^ab^	3.12^ab^	2.21^b^	4.35^a^	4.14^a^	3.43^b^	3.29^b^	2.20^c^	4.09^a^	4.12^a^
SD	0.23	0.07	0.09	0.55	0.72	0.04	0.17	0.02	0.15	0.01
LEU*	6.63^ab^	5.50^bc^	3.80^c^	7.63^a^	7.95^a^	6.06^b^	6.01^b^	3.76^c^	7.04^a^	7.27^a^
SD	0.39	0.19	0.17	0.92	0.19	0.03	0.31	0.01	0.22	0.25
LYS*	8.02^aY^	5.75^bZ^	4.20^c^	7.61^a^	6.84^ab^	6.71^bZ^	6.50^bY^	4.39^c^	8.23^a^	6.32^b^
SD	0.41	0.02	0.19	0.46	0.26	0.11	0.21	0.04	0.19	0.69
MET*	2.55^ab^	2.05^bc^	1.74^c^	2.89^a^	2.26^abc^	2.40^b^	2.14^c^	1.73^d^	2.72^a^	2.37^b^
SD	0.13	0.05	0.04	0.32	0.13	0.01	0.01	0.01	0.05	0.01
PHE*	3.73^ab^	3.32^b^	2.50^c^	4.13^a^	4.36^a^	3.50^b^	3.58^b^	2.48^c^	4.04^a^	4.26^a^
SD	0.14	0.10	0.08	0.36	0.23	0.02	0.14	0.01	0.08	0.09
PRO	5.69^b^	8.03^ab^	10.02^aZ^	4.28^b^	5.96^b^	7.11^b^	7.66^b^	10.97^aY^	5.72^c^	6.29^c^
SD	1.46	0.81	0.20	1.35	0.60	0.05	0.32	0.11	0.28	0.09
SER	4.58^b^	4.48^b^	4.34^b^	4.60^b^	5.19^a^	4.47^b^	4.54^b^	4.14^c^	4.46^b^	5.07^a^
SD	0.14	0.12	0.01	0.13	0.02	0.05	0.03	0.04	0.02	0.05
THR*	4.38^a^	3.98^ab^	3.13^b^	4.79^a^	4.98^a^	4.14^b^	4.06^b^	3.09^c^	4.52^a^	4.83^a^
SD	0.09	0.08	0.06	0.37	0.43	0.03	0.16	0.00	0.08	0.07
TYR	2.92^ab^	2.42^bc^	1.37^c^	3.34^ab^	3.73^a^	2.67^c^	2.71^c^	1.42^d^	3.22^b^	3.88^a^
SD	0.15	0.06	0.10	0.48	0.40	0.01	0.16	0.03	0.11	0.05
VAL*	4.55^abY^	4.29^bc^	3.38^c^	5.09^ab^	5.34^a^	4.25^bZ^	4.21^b^	3.25^c^	4.66^a^	4.85^a^
SD	0.05	0.17	0.08	0.46	0.20	0.04	0.14	0.00	0.12	0.08
EAA	35.46^ab^	29.76^b^	22.13^c^	38.45^a^	38.13^a^	32.37^b^	31.71^b^	22.21^c^	37.35^a^	36.28^a^
SD	1.50	0.62	0.74	3.65	1.82	0.09	1.13	0.04	0.92	0.15
NEAA	64.54^bc^	70.24^b^	77.87^a^	61.55^c^	61.87^c^	67.63^b^	68.29^b^	77.79^a^	62.65^c^	63.72^c^
SD	1.50	0.62	0.74	3.65	1.82	0.09	1.13	0.04	0.92	0.15

Values are means from two replicate samples and standard deviation.

EAA is essential amino acids (*), NEAA is nonessential amino acids.

a, b, c, d within a row and within species (channel or Hybrid) represent significant differences (*p* < .05) based on Tukey‐Kramer adjustment to least square means test.

Y, Z within a row but between common byproduct parts (channel frames vs. hybrid frames) represent significant differences (*p* < .05) based on Tukey‐Kramer adjustment to least square means test.

Amino acid analysis of skins indicated substantially higher levels of glycine and proline than other byproducts and, in addition, skin contained lower amounts of the sulfur‐containing amino acids methionine. Nagai and Suzuki (2000) have reported the distinctive amino acid profile of collagen. The hydroxyproline content found in channel and hybrid heads was 5.00% and 4.16%, respectively. Low levels of hydroxylysine were detected in all tissues and the content was greater in hybrid byproducts than comparable channel catfish byproducts parts. Arginine content of byproducts ranged from 6.7 to 8.1%.

### 3.4 Geosmin and MIB determinations

Table 4 lists the concentrations of geosmin and MIB that were determined in the channel and hybrid byproduct parts. MIB was not detected in any of the byproduct samples analyzed. The MIB assay detection limit was greater than 0.01 ppb. Geosmin was detected in all channel and hybrid byproducts; however, the concentrations were all below 1 ppb. The highest concentration was in hybrid fillet mince (0.9 ppb), which had a lipid content of 21.6% and the lowest concentration of geosmin was in hybrid skin (0.24 ppb), which had a low fat content of 10% (Table 1). There is little indication of MIB or geosmin being sequestered in any of the byproduct tissues examined. The sensory threshold for detection of geosmin in farm‐raised barramundi has been reported to be 0.74 ppb (Jones, Fuller, & Carton, 2013), and 0.9 ppb in farm‐raised trout (Robertson, Jauncey, Beveridge, & Lawton, 2005).

**Table 4:** Concentration (ppb) of geosmin and MIB in channel and hybrid catfish byproducts

	Channel					Hybrid				
	Frame	Head	Skin	Mince	Viscera	Frame	Head	Skin	Mince	Viscera
MIB	N/D	N/D	N/D	N/D	N/D	N/D	N/D	N/D	N/D	N/D
GSM	0.31^bZ^	0.28^b^	0.45^ab^	0.77^a^	0.41^ab^	0.54^bY^	0.31^bc^	0.24^c^	0.90^a^	0.31^bc^
SD	0.12	0.06	0.24	0.12	0.23	0.04	0.11	0.02	0.11	0.16

Values are means from three replicate samples and standard deviation.

MIB is 2‐methylisoborneol.

a, b, c, d within a row and within species (channel or Hybrid) represent significant differences (*p* < .05) based on Tukey‐Kramer adjustment to least square means test.

Y, Z within a row but between common byproduct parts (channel frames vs. hybrid frames) represent significant differences (*p* < .05) based on Tukey‐Kramer adjustment to least square means test.

N/D = not detected (<0.01 ppb).

## 4 Conclusion

Comparable channel and hybrid byproducts are similar in composition including % moisture, lipid, ash, protein, and fatty acid and amino acid profiles. There were major differences in the composition of different byproduct parts with the skin being the most different with a lower lipid content and a distinctive amino acid profile. All channel and hybrid byproducts had high % lipid values between 9.3% and 21.6%. The catfish off‐flavor compound, geosmin, was present in all byproduct samples at concentrations below 1 ppb. Results from this study will be used in the development of new value‐added products from catfish byproducts.

## Conflict of Interest

None declared.
